# Military Service Member and Veteran Reintegration: A Conceptual Analysis, Unified Definition, and Key Domains

**DOI:** 10.3389/fpsyg.2017.00369

**Published:** 2017-03-14

**Authors:** Christine A. Elnitsky, Michael P. Fisher, Cara L. Blevins

**Affiliations:** ^1^College of Health and Human Services, University of North Carolina at CharlotteCharlotte, NC, USA; ^2^College of Medicine, The University of CincinnatiCincinnati, OH, USA; ^3^Health Psychology Program, University of North Carolina at CharlotteCharlotte, NC, USA

**Keywords:** veterans, military, reintegration, coping, adjustment, deployment

## Abstract

Returning military service members and veterans (MSMVs) may experience a variety of stress-related disorders and challenges when reintegrating from the military to the community. Facilitating the reintegration, transition, readjustment and coping, and community integration, of MSMVs is a societal priority. To date, research addressing MSMV reintegration has not identified a comprehensive definition of the term or defined the broader context within which the process of reintegration occurs although both are needed to promote valid and reliable measurement of reintegration and clarify related challenges, processes, and their impact on outcomes. Therefore, this principle-based concept analysis sought to review existing empirical reintegration measurement instruments and identify the problems and needs of MSMV reintegration to provide a unified definition of reintegration to guide future research, clinical practice, and related services. We identified 1,459 articles in the health and social sciences literature, published between 1990 and 2015, by searching multiple electronic databases. Screening of abstracts and full text review based on our inclusion/exclusion criteria, yielded 117 articles for review. Two investigators used constant conceptual comparison to evaluate relevant articles independently. We examined the term reintegration and related terms (i.e., transition, readjustment, community integration) identifying trends in their use over time, analyzed the eight reintegration survey instruments, and synthesized service member and veteran self-reported challenges and needs for reintegration. More reintegration research was published during the last 5 years (*n* = 373) than in the previous 10 years combined (*n* = 130). The research suggests coping with life stresses plays an integral role in military service member and veteran post-deployment reintegration. Key domains of reintegration include individual, interpersonal, community organizations, and societal factors that may facilitate or challenge successful reintegration, and results suggest that successful coping with life stressors plays an integral role in post-deployment reintegration. Overall, the literature does not provide a comprehensive representation of reintegration among MSMVs. Although, previous research describes military service member and veteran reintegration challenges, this concept analysis provides a unified definition of the phenomenon and identifies key domains of reintegration that may broaden our understanding and guide reintegration research and practice.

## Introduction

Since 2001, nearly 3 million U.S. military service members have deployed to Operation Enduring Freedom (October 2001–present), Operation Iraqi Freedom (March 2003–August 2010), or Operation New Dawn (September 2010–December 2011). Formerly deployed military service members and veterans (MSMVs) report a high prevalence of physical and mental health problems including post-traumatic stress disorder (PTSD), traumatic brain injury (TBI), anxiety, major depression, and difficulty transitioning from their military operations to civilian roles (summarized in Elnitsky et al, 2013; Sayer et al., 2014). Even among veterans without physical or psychological disorders, research has shown that 25% or more report difficulty in social functioning, self-care, or other major life domains following deployment (Sayer et al., 2011). Many veterans experience relationship and employment difficulties (Sayer et al., 2011), homelessness, post-deployment injury, or suicide (IOM, 2010; Bachynski et al., 2012). Furthermore, the suicide rate among MSMVs exceeds the rate among civilians (Kuehn, 2009; Levin, 2009). Therefore, helping these veterans to resume participation in their life roles is a national priority (U. S. Department of Veterans Affairs [DVA], 2010). Based on our review of over 15 years of research literature on reintegration, we define *MSMV reintegration* as both a process and outcome of resuming roles in family, community, and workplace which may be influenced at different levels of an ecological system. The current article describes the systematic approach through which this definition emerged, while a previous article describes the critical analysis of the literature on reintegration (Elnitsky et al., 2017).

The empirical literature on health and social services is filled with references to reintegration. Some authors have discussed MSMV reintegration after deployment to Iraq and Afghanistan. Others have asked whether veterans who return with TBI or mental health issues have hope of reintegrating to productive civilian roles in the community. The goal of health and social services is to improve reintegration. However, the meaning of reintegration may differ when applied by scholars and practitioners to different problems. Does reintegration refer to health status, employment, family relationships, or some combination of these and other factors? Often, authors do not define the concept, leaving readers unclear about the term and their conceptualization of the problems and related factors. Further complicating the understanding of reintegration is the frequent use of potentially overlapping terms such as transition, readjustment, and community integration.

### Conceptualizing reintegration

Reintegration has previously been defined as “the resumption of age, gender, and culturally appropriate roles in the family, community, and workplace” (U. S. Department of Veterans Affairs [DVA], 2010, p. 1) and the process of transitioning back into personal and organizational roles following deployment (Currie et al., 2011). Furthermore, reintegration has been described as a dynamic process of adapting that is culturally bound, personal, and multidimensional (Reistetter and Abreu, 2005). Community reintegration has been described as the return of individuals to their role functions or participation in life roles (Resnik et al., 2012). Although, reintegration is often conceptualized as a positive series of events, it also maybe a time of personal stress and difficulty for MSMVs. A review of the literature suggests that the period following a return from deployment may be associated with increased tension at the personal, family, and work levels, and exacerbation of deployment-related stress conditions (Bolton et al., 2008).

A number of theoretical frameworks have been presented in the literature that focus on transition in an attempt to explore processes of MSMV reintegration. Schlossberg's (1995) theory of transition posits that the individual MSMV's situation, self, support, and strategies facilitate or impede successful transition (Robertson, 2013; Schiavone and Gentry, 2014). An alternate model of MSMV transition (Adler et al., 2011b) suggests that the effect of deployment-related factors on physical, emotional, and social domains of transition are moderated by the psychological processes involved in decompression (i.e., returning from the battlefield to a normal atmosphere), unit variables (i.e., leadership quality, cohesion), and the anticipation of redeployment. Other studies have employed multidimensional theories of grief (Kaplow et al., 2013), relational turbulence (Theiss and Knobloch, 2013), and engagement in diverse aspects of participation (Resnik et al., 2012) in their attempts to explain MSMV reintegration. However, across these studies the lack of a common definition for reintegration has led to inconsistencies in the understanding of what constitutes reintegration and makes it difficult to measure reintegration reliably and conduct research on the topic. This fragmentation limits the potential impact of the MSMV research and its ability to guide practice.

The purpose of this concept analysis is to clarify the meaning of MSMV reintegration. In the process, we determine a unified definition of reintegration, analyze current reintegration measurement instruments, and identify MSMV-reported needs and challenges to reintegration. Ultimately, we aim to further understand the concept and the systems that contribute to reintegration. Using a principle-based concept analysis approach (Penrod and Hupcey, 2005) we explicate the meaning of the concept *reintegration*, focusing exclusively on use of the concept in scientific literature, compare the terms used for the concept over time, and examine reintegration surveys and MSMV reintegration needs. The results of this analysis will enhance understanding of reintegration for MSMVs and inform the development of health and social services.

First, we analyze use of the concept *reintegration* in the literature, differentiating it from the related concepts of transition, readjustment, and community integration as it relates to MSMVs' lives. We then analyze instruments that measure reintegration and synthesize MSMV's self-reports of reintegration challenges and needs, in order to identify what the concept is and is not. Grounded in the empirical evidence, this concept analysis provides a unified definition of the phenomenon and identifies key domains of reintegration that may guide reintegration research and practice.

## Methods

### Search strategy and data collection

Empirical articles were identified in leading databases: Academic Search Complete; Anthropology Plus; ArticleFirst; ERIC; GPO Monthly Catalog; MEDLINE; WorldCat; and WorldCat.org. The search included the following constraints: (a) terms and phrases that included reintegrat^*^, re-integrat^*^, transition^*^, community integration, integrat^*^ in^*^ the community, readjust^*^; (b) sources using the above terms in combination with either the term veteran^*^ or military; and (c) articles published in English between 1990 and June 2015. The scope of the search was deliberately broad to include definitions from various perspectives and to identify trends in terminology arising in the current era. These searches yielded 1,459 articles. We eliminated duplicates and read abstracts and articles to determine if they met the following inclusion criteria: (a) published in a peer-reviewed journal; (b) focused on U.S. MSMV population issues; (c) focused on health or social issues related to returning from war. Applying these criteria and removing duplicates yielded 466 articles for review. Of these, 213 studies used *reintegration* or a related term in the body of the text; 96 studies that used the term only in a reference list, appendix, or title were excluded from the study. Screening of abstracts and full text review based on our inclusion/exclusion criteria, yielded 117 articles for review.

### Analysis process

Characteristics of each article were abstracted and coded into a database by two authors using a data coding dictionary and evidence table developed and revised to include details of the studies (Galvan, 2014). Three interrelated coding processes were applied to each article to address the multiple aims of our analysis. First, a qualitative approach using a comparative analysis grounded in the evidence was adopted (Corbin and Strauss, 2008) to conduct the concept analysis of *reintegration* and related terms (i.e., transition, readjustment, and community integration). To define the boundaries of *reintegration*, each article was reviewed and phrases containing the related terms were extracted and analyzed to determine what the terms did and did not describe. Reviewing all the paragraphs containing the term *reintegration*, we constructed categories depicting the various ways reintegration was used (Corbin and Strauss, 2008), whether involving explicit description (e.g., reintegration is associated with family function) or implied meaning. We also examined articles addressing challenges and needs self-reported by MSMVs in this sample of articles. Each article's *reintegration* definition (if provided) and operationalization of the reintegration concept was coded. In addition, each article was coded to identify factors associated with reintegration which were then grouped into major themes or domains. Codes and themes “emerged” from the literature as we set out to discover the definition implicit in the data. We also counted the annual use of reintegration and related terms across articles to provide a historical picture of the terminology used. Data were abstracted by one investigator and reviewed for accuracy by one additional investigator.

Second, we described measurement instruments using coded data, specifically study design, setting, number and type of subjects, sample selection, and domains of measurement. Finally, we examined articles that addressed challenges and needs self-reported by post-9/11 MSMVs in our sample of articles. We classified these articles by number and type of subjects, key findings, and categories of reintegration needs addressed.

Our research team synthesized results from our analyses to identify a unified definition and key domains of reintegration. Specifically, we considered our analysis of the reintegration concept, existing measurement tools, and MSMV self-reported challenges and needs to determine the breadth of factors, or key domains, typically associated with reintegration. Regular discussions among the investigators facilitated this process. This systematic approach allowed us to reach conclusions about the concept of reintegration and related domains that were grounded in the evidence. Our inclusion of veterans' self-reported challenges and needs helped us to avoid the potential bias of using exclusively researcher-based perspectives.

## Results

### Mapping the use of the reintegration concept and related terms

**Figure 1:**
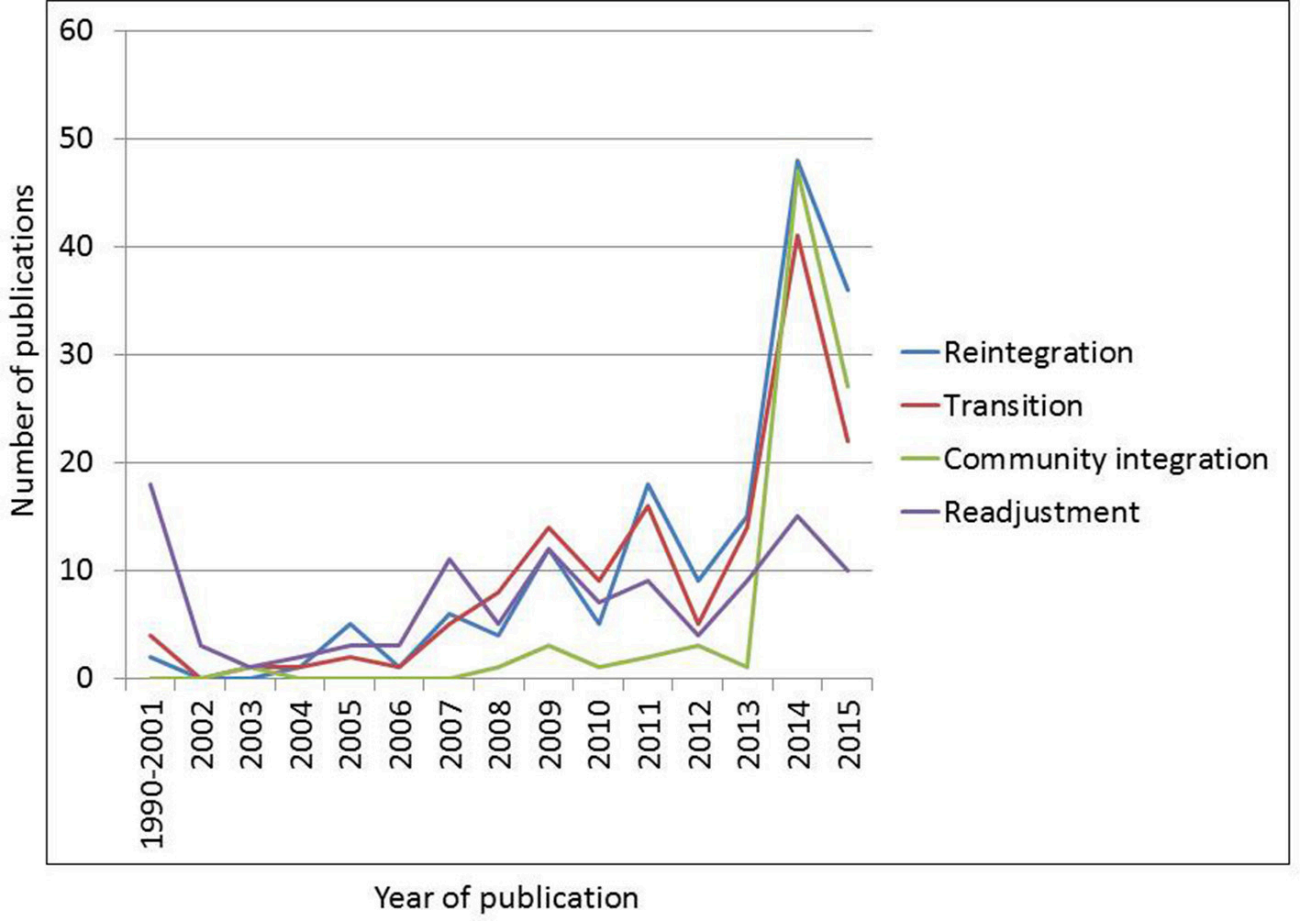
**Trends in reintegration and related term use 1990–2015**.

To analyze and synthesize the term *reintegration* as used in the health and social sciences literature, we reviewed related terms that often overlap with or are used interchangeably with reintegration. These related terms, as well as reintegration itself, are described below. As Figure 1 indicates, there has been greater than a four-fold increase in peer reviewed literature on reintegration and its related terms over the past 5 years.

The term *reintegration* is relatively new in the scientific literature on MSMV's. The term was seldom used before 2004 when its use began to increase dramatically (see Figure 1). The term was used more in 2011 than in any other year, perhaps reflecting the rapid withdrawal of U.S. troops from Iraq and the scientific community's recognition of their needs.

Use of the term *transition* has followed a similar pattern, becoming increasingly common around 2004. However, unlike *reintegration* and *transition*, the term *readjustment* has been used in the scientific literature for more than two decades. Its use has decreased since 2009 though it is unclear why; it may have fallen out of favor as the term *reintegration* gained traction. Alternatively, the scientific community may be placing more emphasis on the various domains of life in which psychological, physical, or social functioning may be improved, and is adopting the term *reintegration* because of its emphasis on multiple domains. *Community integration*, unlike the other terms included in this analysis, has been used rather infrequently over the past decade.

**Figure 2:**
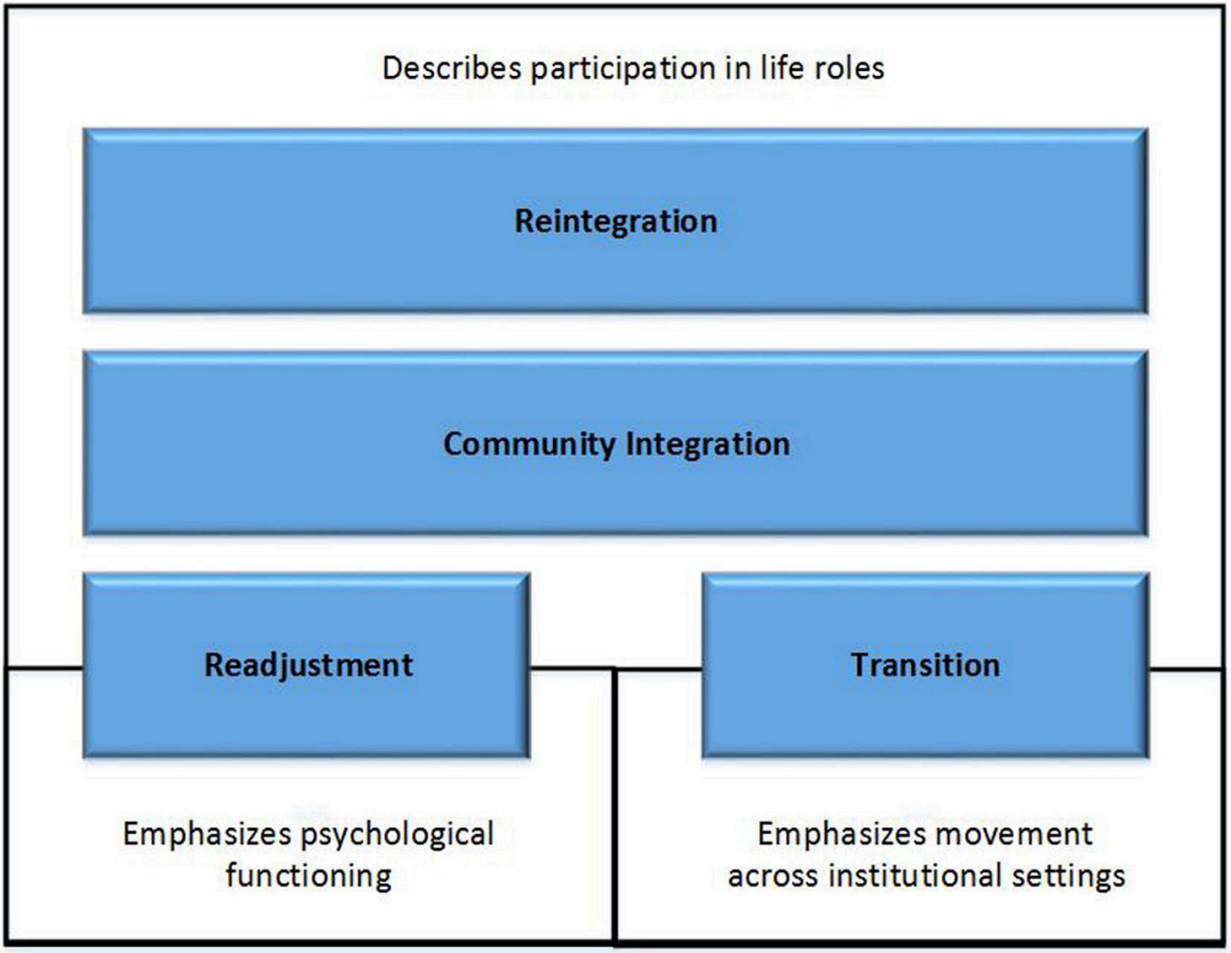
**Primary distinctions between reintegration and related terms**.

*Reintegration* and related terms describe a time period, process, or outcome that MSMVs may experience following military service. Figure 2 shows that both *reintegration* and *community integration* place primary emphasis on participation in life's many roles—as an employee at work (Drebing et al., 2007; Brown, 2008), a student at school (Ackerman and DiRamio, 2009; Bauman, 2009; DiRamio and Spires, 2009; Baechtold and Danielle, 2011), or a spouse (Cohan et al., 2005) or parent within one's family (Grantz, 2007; Chandra et al., 2010). *Readjustment* and *transition* also describe participation in life roles; however, they tend to highlight specific phenomena. *Readjustment* tends to emphasize psychological functioning, that is readapting to civilian life after deployment (Gironda et al., 2009; Sayers et al., 2009), while *transition* tends to emphasize movement across institutional settings—for example, return from deployment (e.g., transition from warrior to civilian; Greene et al., 2010), separation from a military setting and movement to a civilian setting (Glover-Graf et al., 2010; Penk et al., 2010) or from one health care setting to another (Scherrer et al., 2014).

#### Transition

The term *transition* generally refers to either the time period or process during which a MSMV moves from a military to a civilian setting (Rosenheck et al., 2003; Bolton et al., 2008; Casarett et al., 2008), or the movement through various systems of health care (Malphurs and Striano, 2001; Kasprow and Rosenheck, 2007). Phrases such as “transition to veteran status” are common and tend to emphasize movement into or across institutional systems such as the Department of Veterans Affairs. *Transition* is also used to describe services or sets of services provided to MSMV's. However, given the range of possible needs and services, the specific meaning of “transitional services” is unclear.

#### Readjustment

*Readjustment* refers to the process of readapting to civilian life after deployment (e.g., Wolfe et al., 1993; Katz et al., 2007). Like the term *transition, readjustment* pertains to shifting from a military to a civilian role; however, *readjustment* typically evokes images of the MSMV grappling with psychological or emotional issues, including PTSD. The term has been used to refer to psychological health and social issues in a wide range of life roles including work, education, interpersonal relationships, and health (e.g., marital, family, or financial difficulties, homelessness, work issues, medical problems, and motor vehicle accidents). *Readjustment* has also been used in service program titles such as Readjustment Counseling Service—U.S. Veteran Compensation Programs, 2015 and to refer to education and economic benefits such as the Servicemen's Readjustment Act of 1944—also known as the G.I. Bill. Another common use of the term is in the National Vietnam Veterans' Readjustment Study (NVVRS) of the prevalence of PTSD among Vietnam veterans (see Kulka et al., 1990 for review). It is also used frequently without specific reference to what the term covers (e.g., “readjustment to civilian life”).

#### Community integration

Like the terms *transition* and *readjustment, community integration* pertains to separation from the military or return from deployment (Nidiffer and Leach, 2010). It is sometimes used in the context of physical rehabilitation (e.g., “the community integration of severely wounded veterans”; Taylor et al., 2003; Sporner et al., 2009; Wehman et al., 2009). The term *community integration* is used occasionally without description of its meaning.

#### Reintegration

*Reintegration*, the most frequently used term describing separation from the military or return from deployment, often refers to MSMVs' return to the social (e.g., Johnson et al., 1994) or occupational roles (Ortega and Rosenheck, 2000) they filled prior to deployment, however a specific definition is often not provided. *Reintegration* refers to co-occurring psychological, social, health-related, and community-related modes of functioning with one's immediate veteran friends, family, and larger social groups. For example, family -, social-, or community-reintegration, reintegration into society, and reintegration into community life refer to healthy functioning of MSMVs. *Reintegration* also refers to physical rehabilitation needs and systems of care (e.g., *reintegration and rehabilitation* treatment plans, case management, and community-based or in-home rehabilitation services for TBI or polytrauma) as well as employment programs. For example, the Yellow Ribbon Reintegration Program (YRRP), a Department of Defense program was established by U.S. Congress (2008) (Public Law 110–181 Section 582) to help MSMVs reintegrate with communities and employers. Finally, reintegration may also refer to part of military readiness such that active duty personnel are well prepared to deploy repeatedly (e.g., Enhanced Reintegration Action Plan program at Ft. Lewis).

### Domains of reintegration

Overall, 79 articles included explicit definitions of the reintegration concept including the following different dimensions and respective number of articles: (a) psychological health (*N* = 43); (b) family (*N* = 36); (c) physical health (*N* = 29); (d) employment (*N* = 26); (e) housing (*N* = 10); (f) financial (*N* = 9); (g) education, legal, spiritual, and non-specific (*N* = 8). These 10 categories are not mutually exclusive, and most articles referenced *reintegration* in more than one category. After analyzing the literature, we determined, importantly, that no single article in the literature included a comprehensive conceptualization of reintegration across various levels of an ecological model (i.e., individual, interpersonal, community systems, and societal), which is a core theme that emerged from this work (see Elnitsky et al., 2017).

The term *reintegration* referred to any number of issues related to successful functioning in various facets of life: (a) *Psychological health*—behavioral, mental, or emotional symptoms or disorders, or psychosocial functioning; (b) *Social*—interaction with family members, friends, parental or marital relationships, marital issues; (c) *Physical health*—disease, illness, or injury, or wellness; (d) *Employment*—post-military unemployment or jobs; (e) *Housing*—homelessness, shelter/accommodations; (f) *Financial*—personal economic issues; (g) *Education*—college, continuing education at school; and (h) *Legal*—unlawful behavior or criminal justice matters; (i) *Spiritual*—religious or spiritual activities or a sense of meaning or purpose in life; and (j) *Non-specific*—functioning (psychosocial, health, or community-related) without explicit reference to other categories.

In summary, *reintegration* is a broad, holistic concept of overall psychosocial functioning that includes psychological and physical health. It spans more health and social services than the other terms included in this analysis, and it frequently emphasizes physical health and rehabilitation issues. Unlike the other terms included in this analysis, *reintegration* sometimes refers to a key component of military readiness and evokes “positive reintegration experiences,” or the ways in which deployment may enhance one's life or perceived meaning in life.

### Reintegration measurement instruments

Eight Reintegration Measurement Instruments and their characteristics are summarized in Table 1. Overall, the instruments measure all the aforementioned conceptual sub-domains of reintegration, except transition (i.e., as movement across different types of healthcare). Despite this limitation, the results show promise for use of surveys to measure reintegration as either a process or outcome in future studies.

**Table 1:** **Selected characteristics of reintegration measurement instruments (*N* = 8)**.

**Concept domain**	**Instrument**	**Description**	**Concept definition**	**Standardization**	**Reliability**	**Dimensions**	**Validity**	**Summary and evaluation**
Transition	Combat-to-Home Transition Scale (C2HTS)—(Adler et al., 2011a)	A 16-item, self-report measure designed to assess experiences of transitioning home among MSMVs. Responses are noted on a five-point Likert scale (1 = strongly disagree to 5 = strongly agree)	Adjustment following combat deployment, including the experience of psychological benefits and the emotional toll of deployment	Active-duty US Army soldiers (96% male) at 4- and 8-months post-combat deployment	The C2HTS has been found to have adequate to high internal consistency with in dimensions with total score α = 0.90 and individual factors ranging from α = 0.71 to 0.83. Test-retest reliability data were not available	The C2HTS represents four distinct factors underlying transition experiences in MSMVs: Benefit, Appreciation, Anger/Alienation, and Guilt/Remorse	Negative and positive aspects were related to and distinct from PTSD symptoms. In addition, negative scale dimensions were correlated with combat experiences	The C2HTS is a valid and reliable measure of transition experiences post-deployment. The measure has been infrequently used and there is little research on the predictive validity
Readjustment	Iraq Readjustment Inventory (IRI)—(Katz et al., 2007)	A 16-item, self-report measure designed to assess experiences assess social readjustment and deployment concerns of women returning from Iraq. Responses are noted on a five-point Likert scale (1 = not at all to 5 = extremely)	Adjusting to life post-deployment, including education, employment, relationships, and familial roles	Women MSMVs who fought in OEF/OIF and were seeking treatment at a VA medical center	The IRI has been found to have high internal consistency in all dimensions except one (Career readjustment) total score: α = 0.89; Social α = 0.87; and Concerns about Iraq α = 0.81. Test-retest reliability data were not available	The IRI represents both three distinct factors: global measure of readjustment, social readjustment, and concerns about Iraq	Military sexual trauma was significantly related to mental health symptoms and readjustment ratings across all domains. Scales were highly correlated (*r* = 0.53–0.79) with objective clinician ratings of participant symptoms	The IRI is a reliable and valid measure of female MSMVs readjustment difficulty. The scale has been used less frequently and there is little research on the predictive validity of the measure
	Post-Deployment Readjustment Inventory (PDRI)—(Katz et al., 2010)	A 36-item, self-report measure designed to extend the IRI and assess MSMVs serving in additional countries and added domains of functioning. Responses are noted on a five-point Likert scale (1 = not at all to 5 = extremely)	Adjusting to life post-deployment, including emotional, mental health, occupational, relationships and interpersonal challenges	MSMVs (85% male) of OEF/OIF	Analyses revealed high internal consistency for the total scale (α = 0.97) and six subscales: α = 0.82–0.92. Test-retest reliability data were not available	The PDRI represents seven domains: global readjustment, career challenges, health concerns, intimate relationship problems, social difficulties, deployment concerns, and PTSD symptoms	The PDRI demonstrated strong convergent validity with the BSI (*r* = 0.82) and PCL-M (*r* = 0.90). Predictive validity revealed readjustment difficulty to be highly associated with MST (*r* = 0.26), being injured (*r* = 0.40), and witnessing death/injury (*r* = 0.23)	The PDRI is an extension of the IRI which includes the addition of several items to assess male MSMVs, MSMVs serving in other countries, and additional domains of functioning related to readjustment. Reliability and validity has been established
Community Integration	Community Integration Questionnaire (CIQ)—(Willer et al., 1994)	A 15-item, self-report measure designed to assess different aspects of community activity of individuals recovering from a traumatic brain injury (TBI)	The opposite of handicap (i.e., to social disadvantage resulting from disability or impairment) with an emphasis on participation of the individual within their environment	Individuals with TBI living in the community	Several studies have examined the psychometric properties of the CIQ, and it has shown high internal consistency and good test-retest reliability, with scores of *r* = 0.91, 0.93, and 0.86, and 0.83 observed for the total CIQ, HI, SI, and PA scores, respectively	The CIQ can be assessed globally, or within three domains: home integration, social integration, and productive activity (i.e., work/school/volunteer)	Evidence for discriminant validity indicates that the CIQ is able to differentiate between patients with TBI and controls, as well as differentiate between TBI survivors with varying degrees of independence	The CIQ was originally developed due to recognition that community integration is a priority during rehabilitation. The CIQ has been validated and used in a wide range of samples and populations with various degree and type of injury. It is valued for its quantitative properties and ease of use. Short forms of the scale are also available
	Community Integration Measure (CIM)—(McColl et al., 2001)	A 10-item, client-centered, self-report measure designed to assess participation and connections of individuals with TBI in the environment. Responses are noted on a five-point Likert scale (1 = always disagree to 5 = always agree)	A function of four factors pertaining to participation and connection with the environment: assimilation (conformity, orientation, acceptance); social support (close and diffuse relationships); occupation (leisure, productivity); and independent living (personal independence, satisfaction with living arrangement)	Three subsamples: individuals with moderate-to-severe TBI, TBI patient family members, and college students	The CIM demonstrated high internal consistency, with total score α = 0.87. Subgroup alpha values were: TBI patients α = 0.83, college students α = 0.78, and family members α = 0.92. Test-retest reliability data were not available	Principal component analysis confirmed a 1-factor structure comprised of the following domains: assimilation, support, occupation and independent living	The CIM was found to have adequate content, criterion, and construct validity, and was able to differentiate between TBI patients and controls. Comparison of the CIM and CIQ revealed associations of *r* = 0.34, indicating that the measures, while related, are distinct	This CIM is a valid and reliable measure of perceived community integration among persons with a mild-to-severe history of TBI. The CIM has been validated and used in a wide range of studies
Reintegration	The Post-Deployment Reintegration Scale (PDRS)—(Blais et al., 2009)	A 36-item, self-report measure designed to assess positive and negative experiences of military personnel following deployment	Process of transition home and decompressing following overseas military service often accompanied by psychosocial stress	Canadian Forces personnel recently returned from an overseas peace support operation	The PDRS demonstrated moderate to high internal consistency, with reliability estimates ranging from α = 0.78 to 0.89. Test-retest reliability data were not available	The PDRS offers the ability to assess positive and negative reintegration experiences across work, family, and personal domains	Discriminant validity was shown between positive and negative aspects of each domain. Predictive validity revealed that high military commitment and job-related affect predicted positive reintegration experiences	The PDRS is a psychometrically reliable and valid measure of post-deployment reintegration. The scale has been used less frequently and there exists little research on the predictive validity of the measure
	Community Reintegration of Service Members (CRIS)—(Resnik et al., 2009)	A multi-dimensional scale designed to assess community reintegration and participation in life roles as defined by International Classification of Health and Functioning (ICF)	Adjustment to life at home and in the community	MSMVs recruited from a VA medical center primary care clinics	The CRIS demonstrated excellent internal consistency, with reliability estimates ranging from α = 0.91 to 0.97. Subsequen*t*-test–retest reliability analyses revealed ICCs >0.6	Three fixed subscales assess extent of participation, perceived limitations, and participation satisfaction	The CRIS demonstrated excellent construct, convergent, and discriminant validity	The CRIS is a comprehensive measure of community reintegration with conceptual integrity, excellent reliability, and construct, convergent, and discriminant validity. The scale has been adapted for computer and telephone use
	The Military to Civilian Questionnaire (M2C-Q)—(Sayer et al., 2011)	A 16-item self-report measure of post-deployment community reintegration difficulty. Responses are noted on a 5-point Likert scale ranging from 0 = No Difficulty to 4 = Extreme Difficulty	Readjusting to mainstream family and community life, fulfilling normal roles and responsibilities, and being and active and contributing member to one group and society as a whole	Stratified, random sample of OEF/OIF combat veterans using VA healthcare	The M2C-Q demonstrated excellent internal consistency (α = 0.95). Test-retest reliability data were not available	Principal component analysis confirmed a 1-factor structure comprised of the following domains: social and health behaviors; interpersonal relationships; productivity; community participation; (self-care; leisure; and perceived meaning in life. Domains related to physical disability were excluded	The M2C-Q demonstrated excellent construct, convergent, and discriminant validity	The M2C-Q is a comprehensive and psychometrically sound measure of reintegration. The scale is novel and has been used less frequently and there is little research on the predictive validity of the measure

*Transition* was measured by only one tool, the Combat-to-Home Transition Scale, which defines and measures the experiences of transitioning home among U.S. military service members (Adler et al., 2009, 2011a). Adler et al. (2011b) defined transition as the adjustment following combat deployment, including the experience of psychological benefits and the emotional toll of deployment.

*Readjustment* has been measured by a number of scales which provide insight into how the concept is used with the current MSVM population; they include the Iraq Readjustment Inventory (IRI, Katz et al., 2007) and the Post-Deployment Readjustment Inventory (PDRI, Katz et al., 2010). The IRI assesses social readjustment and deployment concerns of women returning from Iraq; the PDRI extends the IRI to assess MSMVs serving in additional countries and added domains of functioning: career and intimate relationship challenges, health problems, and PTSD symptoms (Katz et al., 2010).

*Community integration* has been measured by a number of instruments. The Community Integration Questionnaire (CIQ) is a survey of home and social activities, and work or school activities of individuals recovering from a TBI (Willer et al., 1994). The Community Integration Measure (McColl et al., 2001) is a survey of participation and connections of individuals with TBI in the environment, including assimilation, support, occupation, and independent living (McColl et al., 2001).

Researchers have developed three assessment tools to measure *reintegration* explicitly. The Post Deployment Reintegration Scale measures positive and negative experiences of military personnel in work, family, and personal domains (Blais et al., 2009). The Community Reintegration of Service Members (CRIS) measures nine domains of participation (knowledge, general tasks, communication, mobility, self-care, domestic life, relationships, major life areas, and community, social, and civic life) among injured MSMVs. Three CRIS subscales assess participation frequency, perceived limitations, and satisfaction with a list of individual items (Resnik et al., 2009). The CRIS items relate to skills and problem solving; handling stress and multiple daily tasks; movement, driving and using transportation; self-care and caring for others; interpersonal, family and intimate relationships; acquiring, keeping, and terminating a job; making complex economic transactions; maintaining economic self-sufficiency; recreation and leisure; socializing; and maintaining citizenship and a political life (Resnik et al., 2009, p. 92). The Military to Civilian Questionnaire (M2C-Q), measures general difficulty in readjusting to civilian life following deployment (Sayer et al., 2011) by assessing social and health behaviors, specifically interpersonal relationships; productivity at work, school, or home; community participation; self-care; leisure; and perceived meaning in life. It excludes domains related to physical disability (Sayer et al., 2011, p. 664).

### MSMV reintegration challenges and needs

To analyze and synthesize the *challenges and needs* of post-9/11 MSMVs, we reviewed studies of MSMVs' self-reported needs in reintegrating to civilian life (see Table 2).

**Table 2:** **Reintegration needs reported by post-9/11 military service members and veterans**.

**Author**	**Sample (*n*- type)**	**Description of sample**	**Main findings**	**Categories of reintegration needs**
Sayer et al., 2010	1,226 veterans	National stratified sample of Iraq and Afghanistan combat veterans who use VA	25–56% of veterans report difficulty in social functioning, productivity, community involvement, and self-care domains. Almost all were interested in services to help readjust to civilian life	Social functioning and relationships, productivity, community involvement and belonging, health care (physical and behavioral), risky behaviors (i.e., driving, substance use), substance use, anger management, suicidal/homicidal ideation, legal problems and spirituality
Beder et al., 2011	871 veterans, service members	Male and female veterans responding to survey online or in person	Reintegration difficulties varied by exposure to direct combat, being wounded, having PTSD, having multiple deployments, and lengths of deployment 6 months or more, and gender of veteran	Personal (sense of identity), relationships, and productivity (work/school)
Plach and Sells, 2013	30 veterans	Veterans 20–29 interviewed and surveyed in health screenings at university campus for occupation reintegration issues	Top five occupational performance challenges reintegrating to community and daily life were relationships, school productivity, and self-care. Respondents screened positive for most common mental health and brain injuries	Self-care (driving, sleep disruption, finances, physical health, interactions, mental health); productivity, leisure (relationships, drinking, balancing time), and mental health (PTSD, TBI, major depression, alcohol abuse)
Bloeser et al., 2014	152 veterans	Veterans recently separated and coming to a large urban VA Medical Center	Post-deployment difficulties and functional impairments were related to participation in VA mental health care	Problems with school and work, physical fights, physical health problems, financial difficulties, irritability/anger, isolation, drug use, problems with social support
Larson and Norman, 2014	461 recently separated veterans	Recently separated Marine veterans	PTSD symptoms predicted reintegration difficulties across nearly all domains of functioning (other than unlawful behavior). Greater combat exposure increased risk and greater resilience and being married protected against unlawful behavior	Functional difficulties included work related problems, financial problems, unlawful behavior, mental health symptoms limiting activities, post-traumatic stress disorder symptoms
Sayer et al., 2015	1,292 veterans	War veterans responded to a survey and clinical trial of expressive writing	54% prevalence rate of reintegration difficulty; veterans discharged from military 6 years prior. VA users had higher combat exposure, probable PTSD, TBI, distress, physical symptoms, and reintegration difficulty than nonusers	Mental and physical problems, psychological stress, physical symptoms) and difficulties in social, productivity, community or civic engagement, self-care and leisure domains
Wilcox et al., 2015	126 National Guard members	Recently returned from a 1-year deployment in Iraq	Rates of problems were elevated upon return from deployment and remained fairly constant until 6 months post-deployment	Psychological and behavioral problems, relationships, family reintegration challenges

Overall, MSMVs report challenges and needs that may be categorized into a typology of individual, interpersonal, community, and societal issues. Individual challenges and needs reported by MSMVs include physical and psychological health and behaviors (Sayer et al., 2010, 2015; Plach and Sells, 2013; Bloeser et al., 2014; Larson and Norman, 2014; Wilcox et al., 2015), personal identity challenges (Beder et al., 2011), personal spirituality challenges (Sayer et al., 2010), self -care challenges (Plach and Sells, 2013), feelings of isolation (Bloeser et al., 2014), and financial difficulties (Bloeser et al., 2014). Interpersonal challenges reported by MSMVs include difficulties in social engagement (Sayer et al., 2015), social functioning and relationships (Sayer et al., 2010; Beder et al., 2011), lack of social support (Bloeser et al., 2014), and challenges with relationships and family reintegration (Wilcox et al., 2015). Community challenges reported by MSMVs include difficulties with community involvement and belonging (Sayer et al., 2010), and difficulties with productivity in work or school (Beder et al., 2011; Plach and Sells, 2013; Bloeser et al., 2014; Larson and Norman, 2014; Sayer et al., 2015). Societal challenges reported by MSMVs include unlawful behaviors (Sayer et al., 2010; Larson and Norman, 2014) including risky driving (Sayer et al., 2010), and physical fights (Bloeser et al., 2014). Approximately 25–56% of post-9/11 MSMVs experience health, economic, and social challenges (Sayer et al., 2010). Overall, challenges are more common among MSMVs with PTSD, though high proportions of MSMVs experience challenges in multiple dimensions of reintegration, regardless of their mental status.

The typology of key domains of reintegration that include individual characteristics, interpersonal relationships, community systems, and societal needs. These domains represent factors that may hinder successful reintegration of MSMVs or facilitators that may increase the likelihood of reintegration. Community systems and social policy emerged in this analysis as important components impacting reintegration of the MSMV population; the challenges point to the need for health, rehabilitation, education, employment, and legal services.

## Discussion

The purpose of this concept analysis was to provide a unified definition of reintegration, review existing empirical reintegration measurement instruments and identify the problems, challenges and needs of MSMV reintegration to guide future research, clinical practice, and related services. Our review of 15 years of research literature revealed that the term reintegration has been conceptualized differently across empirical studies, measurement instruments, and MSMV self-reported needs. Furthermore, while existing work has largely considered MSMV reintegration from unidimensional, individual perspective, our findings show that reintegration is a multi-dimensional phenomenon influenced by multiple domains as individual factors (e.g., a health condition), interpersonal relationships, community systems (e.g., utilization of a specific service), and societal structures. Thus, *we define MSMV reintegration as both a process and outcome of resuming roles in family, community, and workplace which may be influenced at different levels of an ecological system*.

This definition and the four domains provide clinicians, health and social service organizations, and policymakers a clear concept to inform research and practice, and it lays the foundation to enhance reintegration processes and outcomes. Currently, there is little agreement on how one domain level influences other levels or how the MSMVs' context is best understood. To address this gap in the MSMV reintegration literature, we link findings from a critical review of the literature to a social ecological systems model (see Elnitsky et al., 2017). For example, MSMVs face health, family, employment, and financial issues. These reintegration needs correspond to health and social services provided to assist MSMVs in reintegrating to the community.

### Implications for research

Investigators should seek to understand the various conceptualizations of reintegration, from the basic view of community integration to the most comprehensive conceptualization which considers the impacts of nine dimensions of reintegration (Resnik et al., 2009) on MSMVs' participation in civilian life. Although, different terminologies or uses of terms can highlight varied aspects of reintegration, this variation can lead to misunderstandings based on different conceptualizations and measurements.

Additionally, it is important for researchers to identify the dimensions of reintegration they are using and how this might affect the indicators and measurement methods they use. Likewise, in disseminating research, investigators should seek to define reintegration explicitly and place their research in context of the environment and specific domains of interest.

The multiple domains of reintegration pose opportunities for conceptualizing reintegration across domains (e.g., individual, interpersonal, community, and societal) and selecting indicators and measures specific to those domains. Several indicators have been described in literature, but taken together, none seem to capture the full reintegration concept. Future research must incorporate methods that capture the multiple dimensions of reintegration in order to develop a comprehensive theoretical explanation for the challenges and facilitators related to MSMV reintegration.

Future efforts in reintegration research might also seek to identify the unique contributions of individual, interpersonal, community systems, and societal policy factors that together impact reintegration processes and outcomes. For example, MSMVs frequently experience complex combinations of morbidities (Tanielian et al., 2014) and other challenges to reintegration which require integrated service supports. However there are gaps in service systems which result in a lack of integration of services (U.S. Government Accountability Office [GAO], 2014).

Furthermore, identifying the various domains of reintegration suggests areas for research which would engage multiple disciplines (e.g., rehabilitation, social sciences, epidemiology, psychology, education administration, etc.). First, researchers should consider whether the dimensions described here are addressed in current explanatory and predictive research. Conceptualizing reintegration as complex and multifaceted presents challenges as it will now be viewed as having multiple facets, not just the individual characteristics that have been most studied in the past 15 years. Emerging research highlights interpersonal and societal level factors that warrant further investigation. Second, considering reintegration as a process rather than a steady state will impact factors included in studies. For example, time since deployment is currently considered in studies, but reintegration as a process of change over time brings into question other contextual factors at play during reintegration. Third, reintegration as an outcome has typically been seen as the functional capacity of MSMVs in specific relationships and roles. However, clarification of factors that are part of successful reintegration is needed. Moreover, a broader conceptualization of reintegration as the ability to function across interpersonal, work, school, and other community roles is necessary if we intend to capture reintegration in various contexts.

### Implications for practitioners

Our comprehensive definition of reintegration promotes a broader focus of practice disciplines, underscoring the need to consider the individual MSMV within the broader context of their family, friends, community, and society and recognize that interventions need to attend to influences across key domains of reintegration (i.e., individual, interpersonal, community organizations, and societal factors). For example, MSMVs working to cope with reintegrating to the community may benefit from efforts to build on interpersonal and community strengths and resources to promote health and to create opportunities for peer support and mentoring. Such interventions will facilitate MSMV healthy adaptation and support successful reintegration.

Potential comprehensive strategies could include work to increase education and support services that are responsive to the needs and challenges experienced by MSMVs, including within post-secondary education, work places, and communities, to enhance interpersonal connections and ultimately reintegration. Because the settings and organizations where MSMVs are educated, work and live can support healthy adjustment, transition, and coping, enhancing the strengths, and supports in these environments is critical. These organizations can provide continuing education and professional development events to educate clinical staff and personnel about military culture and reintegration needs. In addition to increasing awareness of the MSMV needs, organizations may plan community events, and social activities necessary for MSMVs to connect with other colleagues and peers. Such activities, including empirically grounded programs, are needed to support a comprehensive response from community systems to the challenges and needs of MSMVs.

In clinical settings, care providers may incorporate screening for occupational health risk exposures of military service within the routine assessment practices of organizations serving MSMVs. While not typically included on assessment intake forms, there is a national “have you ever served” initiative to include screening for such occupational health exposures through identifying if the patient or their family member has ever served in the military as well as incorporating such assessments into the curriculum of health care professional training programs (Collins et al., 2013). Adding this targeted assessment approach will bring community services together and ultimately ensure that our MSMVs have the opportunities, resources, and support they need to reintegrate successfully.

Furthermore, clinicians may apply the unified definition by being aware of the peer connections and social supports that are so important to MSMVs. Clinicians could intentionally identify MSMV relationships and friendships as important resources and help build support to facilitate reintegration. Clinicians may develop and implement Veteran peer-based outreach and treatment groups and assess the overall impacts on MSMV health care engagement and reintegration. Additionally, clinicians may develop innovative programs to support physical and psychological health with a focus on the various interpersonal relationships of individual MSMVs. Providers may implement resilience-building interventions that will enhance relationships and coping strategies and ultimately, reintegration outcomes.

Considering reintegration as a process may help health systems and practitioners identify the most effective timing of interventions to meet MSMV needs and facilitate reintegration. This broader perspective can lead to innovative interventions for the full variety of complex conditions (e.g., psychological, physical, education skills, etc.). Interventions during pre-deployment and deployment phases may promote resilience and reintegration during post-deployment transition. Evaluations of transition across different health care types (e.g., acute care, rehabilitation) and organizations are needed to clarify how these processes influence the reintegration of MSMVs. This view also encourages assessment of barriers in the community, and establishment of multidisciplinary teams to address complex comorbidities and disability and decrease the stigma of psychological health issues in work or school environments. Understanding the challenges and needs of MSMVs and viewing reintegration as having a time element could help clinicians clarify the appropriate timing of interventions as well as the coordination necessary across health and social systems to meet MSMV needs and facilitate reintegration. By applying a multidisciplinary approach to the integrated model of MSMV reintegration (Elnitsky et al., 2017), clinicians could provide for richer perspectives, and diverse intervention approaches to promote MSMV reintegration.

## Conclusions

This article explored the concept of reintegration as it is understood in the context of post 9/11 MSMVs. The authors were motivated by their observation that the meaning of reintegration is currently not comprehensive as indicated by various definitions and lack of consensus on a unified theory of reintegration. This manuscript contributes in-depth understanding of a complex concept, identifying reintegration domains, and relevant levels of consideration using a systematic review of 15 years of peer reviewed empirical evidence.

Current literature points to the need for a unified definition of MSMV reintegration. This definition and the key domains of reintegration combine the perspectives of various disciplines and reflect our current understanding of reintegration, which is expected to continue evolving over time. Applying the unified definition and key domains of reintegration, researchers and practitioners may advance the science on reintegration by including relevant factors at various levels of the model, based on their issue of interest and specific contextual factors.

## Author contributions

Original conception and design of the work CE and MF, substantial contribution to the revised conception and design of the work CB, acquisition, analysis, or interpretation of data for the work CE, MF, and CB, drafting the work CE, MF, and CB, revising the work critically for important intellectual content CE, MF, and CB, final approval of the version to be published and agreement to be accountable for all aspects of the work CE, MF, and CB.

### Conflict of interest statement

The authors declare that the research was conducted in the absence of any commercial or financial relationships that could be construed as a potential conflict of interest.
